# ‘Including health in systems responsible for urban planning’: a realist policy analysis research programme

**DOI:** 10.1136/bmjopen-2015-008822

**Published:** 2015-07-23

**Authors:** Patrick Harris, Sharon Friel, Andrew Wilson

**Affiliations:** 1 Menzies Centre for Health Policy, University of Sydney / Australian National University, Australia; 2 REGNET, Australian National University, Canberra, Australian Capital Territory, Australia

## Abstract

**Introduction:**

Realist methods are increasingly being used to investigate complex public health problems. Despite the extensive evidence base clarifying the built environment as a determinant of health, there is limited knowledge about how and why land-use planning systems take on health concerns. Further, the body of research related to the wider determinants of health suffers from not using political science knowledge to understand how to influence health policy development and systems. This 4-year funded programme of research investigates how the land-use planning system in New South Wales, Australia, incorporates health and health equity at multiple levels.

**Methods and analysis:**

The programme uses multiple qualitative methods to develop up to 15 case studies of different activities of the New South Wales land-use planning system. Comparison cases from other jurisdictions will be included where possible and useful. *Data collection* includes publicly available documentation and purposively sampled stakeholder interviews and focus groups of up to 100 participants across the cases. The units of analysis in each case are institutional structures (rules and mandates constraining and enabling actors), actors (the stakeholders, organisations and networks involved, including health-focused agencies), and ideas (policy content, information, and framing). *Data analysis* will focus on and develop propositions concerning the mechanisms and conditions within and across each case leading to inclusion or non-inclusion of health. Data will be refined using additional political science and sociological theory. Qualitative comparative analysis will compare cases to develop policy-relevant propositions about the necessary and sufficient conditions needed to include health issues.

**Ethics and dissemination:**

Ethics has been approved by Sydney University Human Research Ethics Committee (2014/802 and 2015/178). Given the nature of this research we will incorporate stakeholders, often as collaborators, throughout. We outline our research translation strategies following best practice approaches.

**Strengths and limitations of this study**

- The design allows investigating the complex public health policy problem of engaging in public policy making across another sector in real time.
- The protocol combines innovative realist approaches with more established case study methods and political science frameworks.
- The research develops policy-relevant propositions about the ways to include health in land-use planning systems under various conditions.
- The real-time and politically sensitive nature of the research may lead to difficulty in accessing stakeholders as informants.
- The research is necessarily contextually bounded to New South Wales, Australia.

## Introduction

Extensive evidence linking multiple sectors activities to health outcomes^1^ means that public health organisations are seeking to influence policy and planning activity in other sectors (for recent examples see refs. 2–4). However, the co-benefits of including population health concerns as a policy issue are not well understood or accepted by other sectors,^5^ ^6^ partly driven by their primary roles in achieving specific other government objectives.^7^ ^8^

The system governing the development of the built environment, land-use planning—sometimes known as ‘Urban Planning’—has for over a decade been of specific interest to health advocates because of its irrefutable health impacts.^9^ ^10^ Extensive evidence demonstrates that the way the built environment is planned and built has a pervasive influence on people's health, including obesity, nutrition, depression and infectious disease, and the equitable distribution of these.^11–13^ However, translating that evidence into policy and practice at multiple levels is complex, under-researched and underdeveloped.^14–17^

The opportunity to investigate the inclusion of health across a non-health system is rare. In this programme we study how, why and the extent to which health is considered in different functions of the land-use planning system in New South Wales (NSW), Australia. Recent developments in NSW provide a unique window for investigating how to influence a whole land-use planning system. A review during 2011–2013 of the legislation and system culminated in the draft bill released in October 2013 including health in 2 of the 11 objectives (“to promote health and safety in the design, construction and performance of buildings” and “to promote health, amenity and quality in the design and planning of the built environment”). This influence comes in part, although this has yet to be investigated, from over a decade of health-focused activity in NSW. Investigating the inclusion of health issues in the development of the NSW land-use planning system, at multiple levels, will provide vital knowledge about what is required to support effective health-focused collaboration with a non-health sector.

As an example of the real-time nature of this research, this particular legislative reform stalled in 2014. The current Planning Minister recently indicated support for revisiting the review without starting the whole process again.^18^ Additionally, the activities which influenced the review, particularly the inclusion of health, influenced another major piece of land-use planning policy, the Sydney Metropolitan Strategy. This regional plan, which includes health as one of the four goals, is being further developed and implemented across six metropolitan subregions, affecting sizeable (as in millions) populations.^19^

The *research questions* are:

- What organisational and procedural processes lead to effective cross-sectoral action for health within the NSW land-use planning system following health being recognised as important in the review of the planning legislation?
- How and why did health come to be incorporated as 2 of the 11 legislative objectives during the 2011–2013 review of the NSW land-use planning legislation and system?
- Following the 2011–2013 review, how, why and to what extent are health-related issues, including health equity, taken up and operationalised in two core components of the land-use planning system: ‘plan-making’ and ‘development assessment of major projects’?

*Specific objectives* of the research programme are to:

- Inform health policy and practice in Australia and internationally by providing evidence of the requirements to influence health being included in the strategic legislative, and policy and planning business of a non-health sector;
- Identify the roles and requirements within the health system to engage effectively with land-use planning to develop healthy built environments;
- Develop and test a framework for understanding effective cross-sectoral action for health within complex and dynamic policy systems;
- Develop and test an analytical framework for evaluating land-use plans for their health impact.

Given the importance of examining the whole of land-use planning as a system and that there is some variation between states in Australia and international jurisdictions, the majority of the case studies are based in NSW. In this way, the evolution of the interactions can be traced and comparisons made within the same planning (and health) system(s). NSW is Australia's most populous state (of around 7.5 million, with 4.6 million in the Sydney Metropolitan Area) and thus, is representative of a large and populous jurisdiction. At the same time, however, the programme does also allow flexibility to include cases from other jurisdictions with which we feel comparison will strengthen the design.

Crucially, this protocol responds to recent calls in the international literature for policy-focused research into public policy activity to include health. The political science literature is considered to be underutilised in efforts to influence the inclusion of health within public policy.^8^ ^20^ ^21^ While there is increasing recognition of the importance of political science approaches in understanding health policy systems,^22^ ^23^ this has not yet been used sufficiently to understand activities to influence public policy to improve health and reduce health inequalities.^24–26^

This research is unfolding and will continue to reflect practice in real time over the next 4 years. Given this, the ‘protocol’ requires iteration and flexibility in terms of its application.^27^ This is typical of both realist and real-world political science analysis, explained later, where the attempt is to link research and practice together locally while also refining and adding to the cumulative knowledge base.^27^ ^28^

## Analytic framework

**Figure 1:**
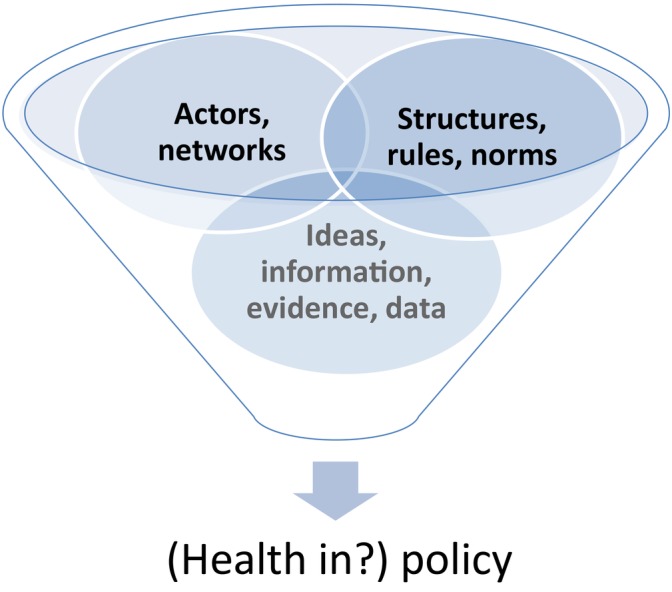
The main elements of policy subsystems which form units of analysis for this research.

Our overarching analytic framework (see figure 1) is adapted from the political science literature regarding the explanation of the influence of policy subsystems on policy processes developed by Howlett *et al*.^29^

Realist methodology investigates and explains complex problems by developing propositions about ‘conditions’ and ‘mechanisms’ which lead to ‘outcomes’ or ‘events’.^27^ ^30^ ^31^ To do this, realist analysis begins by breaking down the problem under investigation into its essential parts.^32^ Essentially, the NSW land-use system has two functions which this research will focus on:^33^ ‘plan-making’, where regional, subregional and local plans are developed; and ‘development assessment’ which is the regulated process of assessing and considering for approval an application for a development project. Additionally, political science theory and research has consistently demonstrated that policy development is rarely linear or rational^28^ but has three core units of analysis as presented in figure 1: *ideas, actors and structures.*^29^ ^34^ These units of analysis will form the basis of explanations about the ‘conditions’ and ‘mechanisms’ which led to the outcome of health being included (and how and to what extent) across the essential aspects of the land-use planning system. A simple example of the analysis for each follows.

‘*Ideas*’ refer to the content of issues in policies, plans and procedures. For health as a cross-sectoral public policy issue there remain definitional tensions—does it refer to ‘hospitals’, ‘illness’, ‘well-being’ or ‘equity’?.^20^ For the business of another sector, the idea of health needs to connect to the substantive issues driving that sector,^35^ for example, the importance of economic development and/or environmental sustainability for land-use planning. We also include the role of information, evidence and data in planning here.

‘*Actors*’ include the stakeholders, organisations and networks^36^ involved in land-use planning: industry, government and regulators, civil society groups, and local communities. Consistent with classic policy analysis theory,^37^ previous research has suggested that policy change principally comes about through learning about health as a relevant issue for the business of another sector.^20^ Different policy actors bring ‘frames’ about specific issues and ideas into the policy arena which, like a picture frame, provide boundaries within which actors value and position their ideas.^38^ Analysis of actors includes the opening of windows of opportunity based on roles, skills and strategies of specific individuals—policy entrepreneurs—in progressing ideas and issues onto policy agendas.^39^ Crucially our focus also includes *the role of and requirements for the health system* when engaging with the land-use planning system. The health system is a vital collaborating partner when another sector considers health and health equity.^7^ ^15^ ^40^ Our focus will be to unpack the organisational requirements, staff competencies and skills, and tools and processes for the health system to collaborate effectively with the NSW land-use planning system.

‘*Structures*’ have several dimensions, including rules and lines of command, divisions of labour, resources, responsibility and channels of communication.^29^ These institutional structures provide the conditions^41^ controlling how or why health may be incorporated or not across the land-use planning system, as well as how the health sector engages with that system. We also include ‘*procedures*’ as crucial structural units for policy.^29^ For example, recent research by PH investigating health input into master planning for urban regeneration found such procedures became important mechanisms for including health, for example, expert advice, specifically commissioned studies, community consultations, checklists, and types of impact assessments.^35^

## Programme of work and methods

The research programme incorporates five overlapping stages of work. Stages 1–3 develop case studies. Stage 4 develops and tests a framework for evaluating land-use plans for their health and health equity impact. Stage 5 compares findings from cases to develop policy-relevant propositions.

### Methods

Stages 1–3 use similar methods. Each develops explanatory *case studies* using qualitative methods following Yin.^42^ The overall conduct of the research is detailed in box 1 against the domains identified in the COREQ checklist for reporting qualitative research.^43^

Box 1 **The conduct of the research against the core domains from the COREQ checklist**

**Domain 1: Research team and reflexivity**

Personal characteristics

- Design and conduct of the research—chief investigators with collaborators relevant to each case study
- Disciplinary backgrounds will vary depending on case studies but are likely to be broad, for example, public health, or urban planning or transport
- Collaborators may be both research partners and evidence users
- Research partners will be engaged in informant identification, analysis and writing, the theoretical interpretation of the empirical data, and will facilitate ownership and translation of the results in their policy-making and practice

Relationship with participants

- Participants likely to be known to chief investigators through prior engagement, or known to the collaborators

**Domain 2: Study design**

Theoretical framework

Realist using political science

- Methods involve mix of content analysis and discourse analysis
- Additional policy and sociological theories will be explicitly searched for where they may offer further explanatory power

Participant selection

- Purposively sampling, with up to 10 informants per case or until the research team agree data saturation has been reached
- Informants will be identified based on their professional engagement with each case, and approached via email or telephone through their professional contact details only

Setting

- Interviews or focus groups will take place in an environment chosen as convenient by the informants
- Only the research team will be present
- Background information collected for each participant to include professional and/or disciplinary background, including length of time working in that field, but only reported such as to maintain confidentiality

Data collection

- Participants provided with interview or focus group guide relevant to phase but with common core analytic dimensions (figure 1)
- Participants able to comment on the guide and specific questions, and told that guide will only be referred to if specific questions or issues have not yet been covered
- The same chief investigator will lead each interview with recorder to make field notes
- Interviews and focus group time limited to 1 h maximum
- Data will be digitally recorded and transcribed. Transcripts will be returned, on request, to participants for comment and/or correction

**Domain 3: Analysis and findings**

Data analysis

- Chief investigators to collaboratively develop, with input from wider research team, initial coding frame—for content and discourse analysis based on figure 1
- NVIVO software to be used to code all data sources—documents or interview/focus group data. Themes will be derived during coding of the data against the coding frame, until the research team agrees data saturation has been reached

Data sources will be coded enabling identification of minor themes or data

A case study is an in-depth study of a single unit, or a group of units, where the researcher's aim is to elucidate features of a larger class of similar phenomena. Case study designs are recognised in public health social science research as providing important insight where other designs (eg, controlled trials) are not possible.^44^ Multiple explanatory case studies focus on how and why phenomena occur, where each case demonstrates or uncovers specific findings which are then either demonstrated or not in other cases.^42^ *Data collection* includes publicly available documentation, including associated media coverage (print and social), and qualitative data collection via purposively sampled interviews with 5–10 participants per case and focus groups, where useful and possible. *Data analysis* will be mostly conducted using NVIVO software (QSR). Content analysis will focus on how ‘health’ is included and conceptualised in documents (eg, as ‘health’ or ‘well-being’ or ‘environmental health’ or ‘health protection’ or ‘health promotion’ or ‘sustainability and health’ or ‘disadvantage’). Interview data will be analysed using a variety of qualitative approaches to develop explanations and propositions about conditions and mechanisms which led to outcomes and events. Realist analysis requires combining concrete and experiential reasoning with abstract, theoretical reasoning.^27^ ^32^ Our suite of analysis, therefore, includes qualitative descriptive analysis which focuses on the data,^45^ and critical discourse analysis which connects the data with theoretically based explanations.^46^ We now describe each of the stages in more detail.

- Stage 1 (2015): How, why and to what extent did health became an objective in the 2011–2013 NSW review of land-use planning legislation?

### Rationale and purpose

This case study research focuses on how health became included in the 2011–2013 review of the NSW land-use planning system and drafting of the legislation. The case being developed is the review itself which includes, but is not limited to, the drafting and passage of the legislation. If this process is revisited or a new activity begins, then additional data will be collected.

- Stage 2 (2015–2017): The extent to which health and health equity concerns are considered in plan-making between 2015 and 2017, and what factors impeded or encouraged this happening.

### Rationale and purpose

The first core function of any land-use planning system, ‘Plan-making’, is the focus of this stage. The planning system emphasises specific state-wide planning objectives and establishes a ‘hierarchy of planning procedures’ to address this: *regional growth plans*, *subregional plans*, and *local plans.*^9^ The Sydney Metropolitan Strategy is an example of plan-making; this regional growth plan is intended to influence subregional plans which, with input from a range of agencies (including health), then influence local environmental plans developed by local governments and through these, to the design of specific local areas. This stage will identify up to six plans—potentially two at each level—covering different regions and locations between 2015 and 2017 to investigate how *health and health equity* are included as a consideration, or not, in the planning and why, including how health as an agency was involved and what this entailed.

- Stage 3 (2015–2018): How, why and to what extent is health included in environmental assessments and approval processes for Major Projects in NSW?

### Rationale and purpose

Development assessment and approval is the second core role of any land-use planning system and is the focus of this stage. This builds on previous content analysis of the coverage of ‘health’ in publicly available ‘environmental assessments’ (EAs) of Major—that is, multimillion dollar investment—proposals in NSW.^11^ This research will investigate this in two ways: content analysis of the inclusion of ‘health’ in a sample of publicly available NSW EA and major project approvals documentation between 2010 and 2018 to identify the extent to which health is considered and whether this has increased over the last decade, and up to six cases of NSW EAs and project approvals.

- Stage 4 (2017–2018): Evaluating plans for their health equity impact

### Rationale and purpose

Assessing and measuring the health equity impact of policies is methodologically challenging because it is rarely possible to have a control community. However, realist evaluation methodologies are now established in public health for evaluating complex programmes.^47^ Between 2017 and 2018, the project will develop and conduct, in collaboration with health and planning stakeholders, an evaluation of up to two specific plans—overlapping with stage 2—for their health equity impact. Informed by findings from stage 2, the evaluation will essentially develop and test a logic model^21^ to identify: (1) policy drivers (eg, economic development, housing) which will impact on health equity; (2) the detail in the plans which will impact on health equity; (3) indicators for outcomes which best represent the health equity effects (both positive and negative) of the plan; (4) the methods to quantitatively and qualitatively measure these effects; and (5) the mechanisms by and conditions through which policy drivers and planning details produce changes in health in the population. This stage overlaps directly with our (PH and SF) National Health and Medical Research Council ‘Centre for Research Excellence on the Social Determinants of Health Equity: Policy Research on the Social Determinants of Health Equity’ (CRE), which has also recently received funding.

- Stage 5 (2018): Qualitative comparative analysis (QCA)

### Rationale and purpose

*QCA* provides an established method for comparing cases for generalisable findings about conditions, mechanisms and outcomes^48^ and developing these as policy-relevant propositions. Up to 15 in-depth cases considering health in land-use planning in NSW (and some comparisons in other Australian and international jurisdictions where this is deemed useful and possible through additional funding sources) would have been developed during this research. QCA is an established method for concisely explaining, using a medium number of cases, causal links between factors under scrutiny while allowing for complexity associated with the conditions that influence these links. The method uses Boolean theory to establish propositions—essentially truth tables—about necessary and sufficient ‘conditions’ and ‘mechanisms’ for an ‘outcome’ to occur across cases.

## Feasibility

The research has two principle feasibility challenges. The research concerns real time, politically sensitive case studies, which will make access to decision-makers and stakeholders challenging. Additionally each case is massive in size and scale, covering large geographic areas as well as populations. These challenges, however, are not insurmountable. Despite the size of the cases, the research draws extensively on publically accessible documentation supported by interviews and focus groups with a manageable number of informants per case. The QCA will be developed with support from an expert in the use of QCA software.

Progress to date demonstrates the feasibility of our approach. We have previously conducted and analysed a purposive sample of documents that informed the review (paper submitted), and conducted 10 stakeholder interviews (including with senior policymakers) and a focus group for stage 1. We are currently developing four cases of major transport infrastructure EAs under stage 3 which will be completed by October 2015 (and which include a comparison case in South Australia). We are also currently identifying plans which have involved health sector inputs to begin developing these in 2016. PH and SF are developing the evaluation framework for stage 4 as part of work for the CRE.

## Dissemination

Findings will be targeted for impact and dissemination in several ways. Given the real-time nature of this research we will incorporate stakeholders throughout, often as collaborators. For example, stage 1 is being conducted as a collaboration between stakeholders across the health and planning sectors, and has resulted in collaboratively writing three conference papers and one paper, with another three papers being planned. A final roundtable will be convened for national and international leaders to discuss the implications of the findings.

The Menzies Centre nodes at the University of Sydney and the Australian National University are conducting leading research and capacity building programmes in health policy and this work will feed directly into that via seminars, coauthoring of journal articles and PhD supervision. Publication through peer-reviewed and grey literature will make the project publicly available. There will be opportunities to incorporate the findings in the set of learning programmes being developed by the Australian Prevention Partnership Centre (AW) and the CRE (SF and PH), both of which use a knowledge-to-action framework. The CRE is comprised of a national policy reference group and an international research translation group through which the findings of this research will be disseminated. Collectively we have connections to policy and practice in the health sector at the three levels of the Australian government (federal, state and local), and in the planning sector at state and local government level. We have cross-disciplinary connections across our institutions locally as well as with national and international universities.
